# Supplementary material for: Motivational Interviewing and Childhood Caries: A Randomised Controlled Trial
Source: Int J Environ Res Public Health. 2023 Feb 27;20(5):4239. doi: 10.3390/ijerph20054239 (PMC10001603; doi:10.3390/ijerph20054239)
Supplement: Supplementary file 1 [file ijerph-20-04239-s001.zip › ijerph-2247606-supplementary.pdf]

Supplementary Figure S1. Self-efficacy, knowledge, attitudes, stress and support scales.

Oral Health Self-efficacy scale; 3 = very confident, 0 = not at all confident
How confident do you feel that you are able to make sure that your child's teeth are brushed before bedtime when you are…
1. Under a lot of stress?
2. Depressed?
3. Anxious?
4. Feeling like you do not have time?
5. Tired?
6. Worrying about other things in your life?
7. Bothered by your child crying?
8. Bothered because your child doesn't stay still when you want him or her to brush?
9. Told by your child that he/she does not feel like brushing right now?
**Knowledge of bottle use scale; 0 = strongly disagree, 4 = strongly agree**
1. Putting a baby to bed with a bottle helps the child to be better fed.
2. Putting a baby to bed with a bottle helps the child sleep better.
3. Putting a baby to bed with a bottle helps the child to gain weight and grow.
4. There is nothing wrong with putting the baby to bed with a bottle.
**Knowledge of children's oral hygiene scale; 0 = strongly disagree, 4 = strongly agree**
1. Cavities in baby teeth don't matter since they fall out anyway.
2. Keeping baby teeth clean is not very important; after all, they fall out.
3. There is not much I can do to stop my child from developing dental cavities.
4. Children don't need to brush everyday until they get their permanent teeth.
5. Children don't really need their own toothbrush until all their teeth come in.
**Oral health fatalism scale; 0 = strongly disagree, 4 = strongly agree**
1. Most children eventually develop dental cavities.
**Parental stress scale; 0 = almost always, 4 = never**
1. How often do you feel that you have too little time to spend by yourself?
2. How often do you wish you did not have so many responsibilities?
3. How often would you say your child (or children) gets on your nerves?
4. How often do you feel that your child/ren is/are making too many demands on you?
5. How often do you feel that being a parent is much more work than pleasure?
6. How often do you feel tired, worn out, or exhausted from raising a family?
**Instrumental support scale, "yes"= 1, "no"= 0**
If you need to, is there someone you can count on to…… 0=No, 1=Yes
1. ……Run errands for you?
2. ……Lend you money?
3. ……Watch over your child/ren?
4. ……Lend you their car or give you a lift?

Supplementary Table S1. Baseline characteristics of children in the test and control group examined at the final follow-up (row percentages* shown).

Baseline Characteristic		Test	Control	P value
Sex; n (%)	Girls=297 (52.5)	153 (51.5)	144 (48.5)	0.76
	Boys=269 (47.5)	142 (52.8)	127 (47.2)	
Location:	Fluoridated=421 (74.4)	218 (51.8)	203 (48.2)	0.78
N (%)	Non-fluoridated=145 (25.6)	77 (53.1)	68 (46.9)	
Education:	≤year 12=90 (16.3)	44 (48.9)	46 (51.1)	0.80
n (%)	Technical/Trade=169 (30.5)	88 (52.1)	81 (47.9)	
	Tertiary=295 (53.3)	156 (52.9)	139 (47.1)	
Family income:	<$AUD80K=231 (41.3)	117 (50.7	114 (49.4)	0.63
n (%)	≥$AUD80K=328 (58.7)	173 (52.7)	155 (57.9)	
Child's Age at follow-up: n=566		n = 295	n = 271	0.64
mean (SD); 5.3 years (0.66)		5.3 years (0.69)	5.3 years (0.64)	
Parent Baby Bottle Knowledge: n=565		n=294	n=271	0.90
mean (SD); 3.1 (3.3)		3.1 (3.4)	3.1 (3.3)	
Parent Baby Oral Hygiene Needs		n=293	n=271	0.24
Knowledge n=564, mean (SD):1.7 (2.1)		1.8 (2.2)	1.6 (2.0)	
Fatalism:	Fatalistic=118 (21.0)	56 (47.5)	62 (52.5)	0.28
n (%)	Non-fatalistic=445 (79.0)	236 (53.0)	209 (47.0)	
Parent self-efficacy: n=563		n=293	n=270	0.79
mean (SD); 18.6 (5.5)		18.5 (5.5)	18.6 (5.5)	
Parental Stress: n=566		n=295	n=271	0.10
mean (SD); 15.8 (4.1)		16.0 (3.6)	15.5 (4.1)	
Parental Support, n (%): n=565				
Errands	Yes=484 (85.7)	260 (53.7)	224 (46.3)	0.05
	No=81 (14.3)	34 (42.0)	47 (58.0)	
Lend money	Yes=502 (89.9)	265 (52.8)	237 (47.2)	0.31
	No=63 (11.2)	29 (46.0)	34 (54.0)	
Babysit	Yes=513 (90.8)	270 (52.6)	243 (47.4)	0.37
	No=52 (9.2)	24 (46.2)	28 (53.9)	
Lend car	Yes=493 (87.3)	261 (52.9)	232 (47.1)	0.26
	No=72 (12.7)	33 (45.8)	39 (54.2)	

*Percentages may not sum to 100 due to rounding.

Supplementary Table S2. Baseline characteristics of children examined at final follow-up and children lost to follow-up, (row percentages* shown).

Baseline Characteristic		Follow-up exam	No follow-up exam	P value
Sex; n (%)	Girls=442 (51.2)	297 (67.2)	145 (32.8)	0.31
	Boys=421 (48.8)	269 (63.9))	152 (36.1)	
Location: n (%)	Fluoridated=657 (76.1)	421 (64.1)	236 (35.9)	0.10
	Non-fluoridated=206 (23.9)	145 (70.4)	61 (29.6)	
Education: n (%)	year12 or less=152 (23.1)	90 (59.2)	62 (40.8)	0.05
	Technical/Trade=262 (29.3)	169 (64.5)	93 (35.5)	
	Tertiary=423 (47.6)	295 (69.7)	128 (30.3)	
Family income: n (%)	<AUD$80K =362 (43.2)	231 (63.8)	131 (36.2)	0.43
	≥AUD$80K =494 56.8)	328 (66.4)	166 (33.6)	
Child's Age at baseline: n=863 mean (SD):	3.2 mths (2.2)	n=566 3.2 (2.1)	n=297 3.2 (2.4)	0.73
Parent Baby Bottle Knowledge: n=862 mean (SD); 3.4 (3.4)		n=565 3.1 (3.3)	n=297 3.8 (3.5)	0.004
Parent Baby Oral Hygiene Needs Knowledge n=861, mean (SD); 1.7 (2.2)		n=564 1.7 (2.1)	n=297 1.7 (2.2)	0.92
Fatalism: n (%)	Fatalistic=196 (22.8)	118 (60.2)	78 (39.8)	0.08
	Non-fatalistic = 664 (77.2)	445 (67.0)	219 (33.0)	
Parent self-efficacy: n=859 mean (SD); 18.2 (5.4)		n=563 18.6 (5.5)	n=296 17.7 (5.3)	0.02
Parental Stress: n=863 mean (SD); 15.8 (3.9)		n=566 15.8 (3.8)	n=297 15.8 (3.9)	0.99
Parental Support, n (%): n=862				
Errands:	Yes=725 (84.1)	484 (66.8)	241 (33.2)	0.09
	No=137 (15.9)	81 (59.1)	56 (40.9)	
Lend money:	Yes=747 (86.5)	502 (67.2)	205 (32.8)	0.009
	No=115 (13.5)	63 (54.8)	52 (45.2)	
Babysit:	Yes=761 (88.3)	513 (67.4)	248 (32.6)	0.002
	No=101 (11.7)	52 (51.5)	49 (48.5)	
Lend car:	Yes=732 (84.9)	493 (67.4)	239 (32.7)	0.008
	No=130 (15.1)	72 (55.4)	58 (44.6)	

*Percentages may not sum to 100 due to rounding.
